# Mapping structural–immune crosstalk in asthma

**DOI:** 10.1093/lifemedi/lnag036

**Published:** 2026-09-24

**Authors:** Lin Chen, Yu-Xuan Jin, Jin-Lyu Sun

**Affiliations:** Department of Allergy, State Key Laboratory of Complex Severe and Rare Diseases, Peking Union Medical College Hospital, Chinese Academy of Medical Sciences and Peking Union Medical College, Beijing 100730, China; Department of Allergy, State Key Laboratory of Complex Severe and Rare Diseases, Peking Union Medical College Hospital, Chinese Academy of Medical Sciences and Peking Union Medical College, Beijing 100730, China; Department of Allergy, State Key Laboratory of Complex Severe and Rare Diseases, Peking Union Medical College Hospital, Chinese Academy of Medical Sciences and Peking Union Medical College, Beijing 100730, China

In a recent study published in *Nature Immunology*, Régis Joulia et al. [1] employed the Xenium high-plex *in situ* transcriptomic platform together with the GeoMX spatial transcriptomics system to generate the most comprehensive spatial cellular atlas of the human asthmatic airway to date. Their work provides a precise delineation of the cellular composition and spatial organization of the bronchial epithelium and lung parenchyma in healthy individuals and patients with asthma, aligning with emerging efforts to chart the spatial architecture and niche reconfiguration of the human lung [2, 3]. The study reveals that airway epithelial cells, mucous gland cells, fibroblasts, endothelial cells, and mast cells collectively form focal inflammatory hubs, in which alarmins such as interleukin-33 (IL-33) and thymic stromal lymphopoietin (TSLP), together with multiple chemokines, create well-defined spatial inflammatory hotspots [4, 5]. Notably, these hotspots are not primarily immune-cell-driven; instead, they are orchestrated by specific structural-cell subsets that establish key microenvironmental units sustaining chronic inflammation and tissue remodeling (Fig. 1).

**Figure 1.**
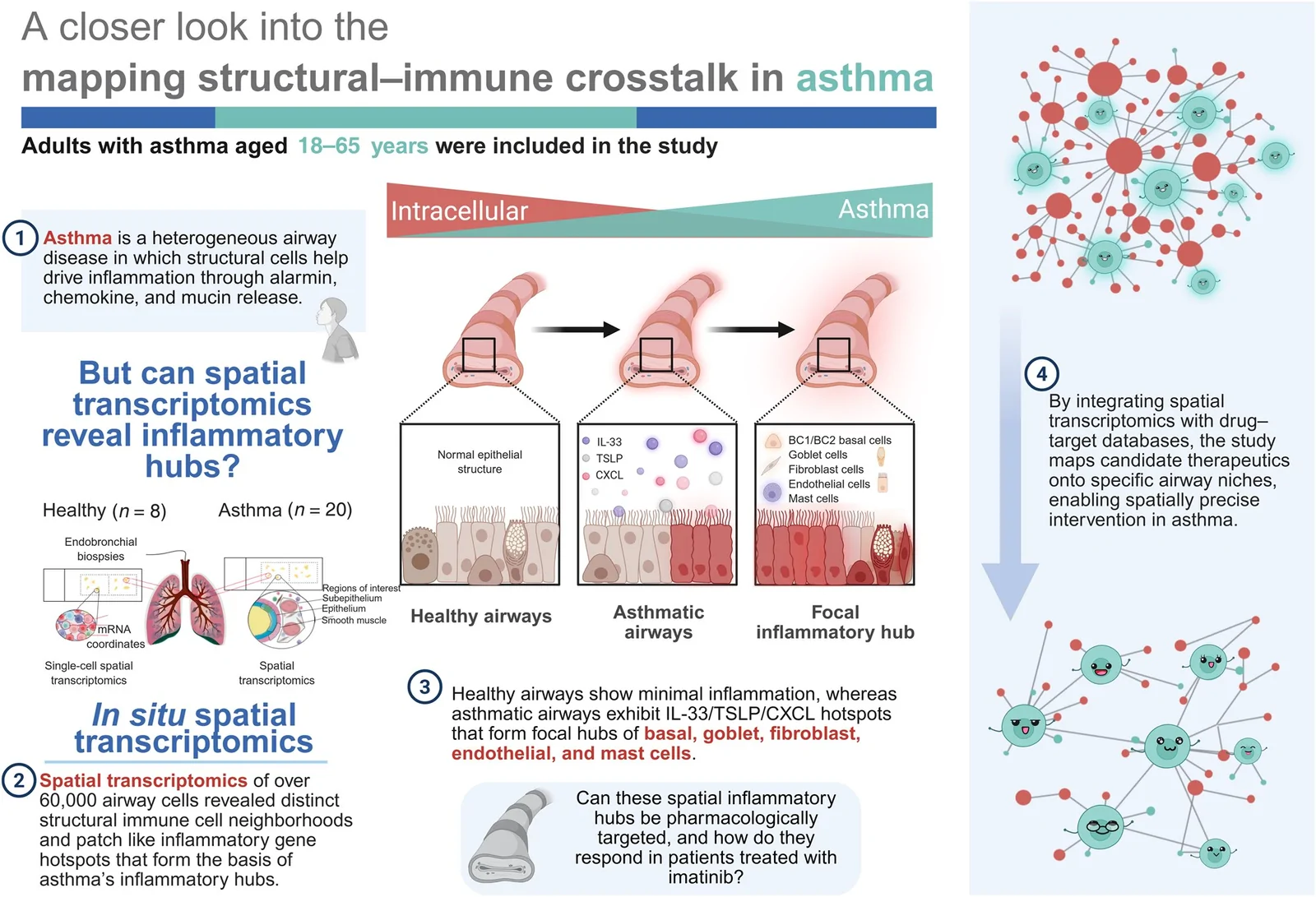
Mapping structural–immune crosstalk and spatial inflammatory hubs in asthma. Asthma is a heterogeneous airway disease in which structural cells contribute to inflammation through the release of alarmins, chemokines, and mucins. Spatial transcriptomics is required to determine whether such inflammatory hubs can be identified *in situ*. Xenium-based spatial transcriptomics of more than 60,000 airway cells from healthy (*n* = 8) and asthmatic (*n* = 20) donors revealed distinct structural–immune neighborhoods and patch-like IL-33/TSLP/CXCL inflammatory hotspots that underlie asthma’s focal inflammatory hubs. Compared with healthy airways that exhibit minimal inflammation, asthmatic airways show epithelial thickening and cytokine-rich hotspots composed of BC1/BC2 basal cells, goblet cells, fibroblasts, endothelial cells, and mast cells. A zoom-in highlights the multicellular architecture of these focal inflammatory hubs. To evaluate their therapeutic tractability, spatial inflammatory hubs were integrated with drug–target databases, revealing candidate agents such as imatinib, gefitinib, and hydrocortisone that map to specific airway niches and may enable spatially precise intervention in asthma. Adapted with permission from Joulia et al. [1], *Nature Immunology*, published by Springer Nature, 2025.

Asthma is a highly heterogeneous chronic airway disorder characterized by persistent inflammation, mucus hypersecretion, and airway structural remodeling. Classical paradigms have emphasized T helper 2 (Th2) cells, group 2 innate lymphoid cells (ILC2s), and eosinophils as the principal drivers of type 2 inflammation. Yet an expanding body of work has shifted attention toward “structural cells”—airway epithelial cells, mucous gland cells, fibroblasts, and endothelial cells—as active organizers of the inflammatory milieu. These cells release alarmins, chemokines, and mucins that shape an inflammation-prone microenvironment. What has remained largely unresolved, however, is a fundamentally spatial question: which cells, *in situ*, constitute bona fide inflammatory hubs through their neighborhood relationships and tissue distribution patterns?

Single-cell RNA sequencing has transformed our understanding of cellular diversity, but it dissociates cells from their anatomical context, erasing the geometry of the airway wall. Addressing this critical gap, Régis Joulia et al. used a spatial multi-omics strategy to chart an *in situ* cell–cell interaction landscape of the asthmatic airway, spanning from tissue-scale architecture to single-cell resolution. By integrating high-plex Xenium *in situ* transcriptomics with GeoMX spatial profiling of bronchoscopic biopsies, registered to matched hematoxylin and eosin (H&E) staining and high-precision cell segmentation, they profile more than 60,000 cells and annotate diverse epithelial, smooth muscle, mucous gland, fibroblast, endothelial, and immune subsets across healthy and asthmatic donors.

A consistent picture emerges from these multi-scale analyses: inflammatory signaling in asthmatic airways is not diffuse but organized into band-like and patchy “inflammatory hotspots.” Within the epithelial and mucous gland niches—two compartments that integrate barrier and secretory function—Xenium transcript-density maps reveal tight co-localization of alarmins (TSLP, IL-33) with multiple chemokine (C-X-C motif) ligand (CXCL) and chemokine (C-C motif) ligand (CCL) chemokines. Dot-plot analyses identify specific fibroblast and endothelial subsets in periepithelial regions that upregulate these mediators, with both the frequency and intensity of positive cells markedly increased in asthma compared with health. At the larger regions of interest (ROI) scale, alarmin signals increase in a stepwise fashion from healthy to mild and severe asthma across epithelial and subepithelial compartments. Together, these findings recast periepithelial stromal and endothelial cells as integral components of localized inflammatory hubs, rather than passive scaffolds that simply host immune cells.

Refined reclustering and spatial statistics further resolve the macroscopic hotspots into discrete cellular neighborhoods. In the epithelial compartment, basal cells (BC1/BC2), goblet cells, and endothelial cells (EnC1/EnC2) emerge as major sources of IL-33 and chemokines, with transcriptional programs enriched for inflammation amplification, barrier regulation, and repair. Nearest-neighbor and shortest-distance analyses demonstrate tight spatial adjacency between specific basal/goblet subsets and EnC2, with local enrichment further accentuated in asthma. GeoMX-based enrichment scoring shows that the abundance and spatial distribution of BC1, goblet cells, and EnC2 are systematically remodeled across epithelial, subepithelial, and smooth muscle regions as disease severity increases. These data support a central basal cell–endothelial cell–goblet cell structural–inflammatory axis as a key organizer of airway remodeling.

Within the mucous glands, goblet and serous cells form a second structural–inflammatory module. Asthmatic goblet cells exhibit markedly elevated expression of mucins such as *MUC5B* and *MUC5AC*, whereas serous cells are enriched for genes related to secretion and innate defense. Spatial neighborhood analyses reveal substantial reconfiguration of goblet–serous relationships and their adjacency to fibroblasts and endothelial cells in asthma, providing a structural framework for mucus hypersecretion, mucus plug formation, and airflow obstruction. Beyond the airway wall, the authors extend their analysis to mast cells in the lung parenchyma. Mast cells show high expression of degranulation- and inflammation-associated genes and spatially overlap with *ACKR1*, *IL33*, and multiple chemokines. Their tight coupling to selected endothelial and stromal subsets positions mast cells as “signal amplifiers” that bridge vascular, stromal, and immune compartments—offering a structural explanation for the coexistence of chronic inflammation and susceptibility to acute exacerbations.

Critically, the study moves beyond descriptive spatial biology. To evaluate the “druggability” of these inflammatory hubs, Régis Joulia et al. analyze Xenium profiles from bronchial biopsies of a clinical trial cohort treated with imatinib or placebo. Across more than 36,000 cells encompassing diverse stromal, epithelial, and immune populations, imatinib treatment significantly reshapes alarmin and chemokine expression in multiple structural and epithelial subsets and alters their spatial adjacency to basal cells, endothelial cells, goblet cells, and mast cells. These observations suggest that tyrosine kinase inhibition can reprogram structural cell–endothelial–immune networks *in situ*, providing preliminary clinical evidence that structural cell-driven inflammatory axes are therapeutically tractable.

Perhaps the most innovative element of the work is methodological: the integration of *in situ* spatial transcriptomic data with the ChEMBL drug–target database to construct a multidimensional drug–cell cluster–spatial location framework. By quantifying potential drug–cell interaction strength and the proportion of targetable cells within each cluster, the authors prioritize candidate agents with high intervention potential in epithelial and mucous gland niches and visualize them through “drug dot plots.” Notably, the visualization reveals an inverse correlation between specific intracellular signaling signatures and asthma status. This suggests that the pathological progression of asthma is characterized not only by the gain of inflammatory mediators but also by the systematic loss of essential homeostatic intracellular regulatory pathways. Such a negative correlation implies that the asthmatic microenvironment actively suppresses endogenous anti-inflammatory or structural integrity-maintaining signals, a finding that further justifies the use of therapeutic agents to pharmacologically restore these depleted intracellular functions. Projecting these drug–target signatures back onto tissue sections generates “spatial action maps” for agents such as tisotumab vedotin, hydrocortisone, imatinib, and gefitinib, highlighting where—within real human tissue—each drug is predicted to act. In doing so, the study begins to shift the conceptual focus from traditional “molecular targeting” toward “spatial targeting,” in which the microanatomical niche becomes a central dimension of therapeutic design.

Looking forward, this spatially grounded framework suggests several priorities for translational research. Functional perturbation of specific basal, endothelial, fibroblast, and mast-cell subsets will be essential to establish causal roles in inflammation amplification, remodeling, and treatment response. Integration of spatial inflammatory patterns with clinical phenotypes, molecular endotypes, and longitudinal outcomes could determine whether niche-based signatures improve risk stratification and therapeutic decision-making. Finally, applying the “spatial omics + drug database” paradigm in larger, multicenter cohorts may accelerate drug repurposing, refine rational combination strategies, and enable truly individualized interventions.

Taken together, the work by Régis Joulia et al. positions asthma as a disease of structural cell-dominated inflammatory hubs and provides a scalable framework that couples high-throughput spatial transcriptomics with drug–target resources. For asthma and other chronic inflammatory diseases, such “spatial precision” strategies offer a path toward therapies that ask not only what to target, but also where in the tissue to intervene—an advance that may ultimately translate into more durable disease control and improved quality of life for patients.
